# Gender as a Modifying Factor Influencing Myotonic Dystrophy Type 1 Phenotype Severity and Mortality: A Nationwide Multiple Databases Cross-Sectional Observational Study

**DOI:** 10.1371/journal.pone.0148264

**Published:** 2016-02-05

**Authors:** Celine Dogan, Marie De Antonio, Dalil Hamroun, Hugo Varet, Marianne Fabbro, Felix Rougier, Khadija Amarof, Marie-Christine Arne Bes, Anne-Laure Bedat-Millet, Anthony Behin, Remi Bellance, Françoise Bouhour, Celia Boutte, François Boyer, Emmanuelle Campana-Salort, Françoise Chapon, Pascal Cintas, Claude Desnuelle, Romain Deschamps, Valerie Drouin-Garraud, Xavier Ferrer, Helene Gervais-Bernard, Karima Ghorab, Pascal Laforet, Armelle Magot, Laurent Magy, Dominique Menard, Marie-Christine Minot, Aleksandra Nadaj-Pakleza, Sybille Pellieux, Yann Pereon, Marguerite Preudhomme, Jean Pouget, Sabrina Sacconi, Guilhem Sole, Tanya Stojkovich, Vincent Tiffreau, Andoni Urtizberea, Christophe Vial, Fabien Zagnoli, Gilbert Caranhac, Claude Bourlier, Gerard Riviere, Alain Geille, Romain K. Gherardi, Bruno Eymard, Jack Puymirat, Sandrine Katsahian, Guillaume Bassez

**Affiliations:** 1 Neuromuscular Reference Center, GH Henri Mondor, AP-HP, Créteil, France, INSERM U955, UPEC university, Créteil, France; 2 INSERM U1138, Centre de recherche des cordeliers, Paris Descartes university, UPMC university, Paris, France; 3 Direction de la Recherche et de l'Innovation, CHU Montpellier, Montpellier, France; 4 Neuromuscular Reference Center, CHU Fort-de-France, Fort de France, France; 5 Neuromuscular Reference Center, CHU Toulouse, Toulouse, France; 6 Neuromuscular Competence Center, CHU Rouen, Rouen, France; 7 Neuromuscular Reference Center, GH Pitié-Salpêtrière, AP-HP, Paris, France; 8 Neuromuscular Reference Center, GH Est, HCL, Bron, France; 9 Neuromuscular Reference Center, CHU Grenoble, Grenoble, France; 10 Neuromuscular Reference Center, CHU Reims, Reims, France; 11 Neuromuscular Reference Center, GH Timone, AP-HM, Marseille, France; 12 Neuromuscular Competence Center, CHU Caen, Caen, France; 13 Neuromuscular Reference Center, CHU Nice, Nice, France; 14 Neuromuscular Reference Center, CHU Bordeaux, Bordeaux, France; 15 Neuromuscular Reference Center, CHU Limoges, Limoges, France; 16 Neuromuscular Reference Center, CHU Nantes, Nantes, France; 17 Neuromuscular Competence Center, CHU Rennes, Rennes, France; 18 Neuromuscular Reference Center, CHU Angers, Angers, France; 19 Neuromuscular Competence Center, CHU Tours, Tours, France; 20 Neuromuscular Reference Center, CHRU Lilles, Lille, France; 21 Neuromuscular Reference Center, Hôpital Marin, AP-HP, Hendaye, France; 22 Neuromuscular Competence Center, HIA Clermont-Tonnerre, Brest, France; 23 PMSI Division, Hox-Com Analytiques Paris, France; 24 CoPil, DM1 patients group, AFM-Téléthon, Evry, France; 25 Human Genetic Research Unit, CHU Laval, Quebec, Canada; University of Valencia, SPAIN

## Abstract

**Background:**

Myotonic Dystrophy type 1 (DM1) is one of the most heterogeneous hereditary disease in terms of age of onset, clinical manifestations, and severity, challenging both medical management and clinical trials. The CTG expansion size is the main factor determining the age of onset although no factor can finely predict phenotype and prognosis. Differences between males and females have not been specifically reported. Our aim is to study gender impact on DM1 phenotype and severity.

**Methods:**

We first performed cross-sectional analysis of main multiorgan clinical parameters in 1409 adult DM1 patients (>18y) from the DM-Scope nationwide registry and observed different patterns in males and females. Then, we assessed gender impact on social and economic domains using the AFM-Téléthon DM1 survey (n = 970), and morbidity and mortality using the French National Health Service Database (n = 3301).

**Results:**

Men more frequently had (1) severe muscular disability with marked myotonia, muscle weakness, cardiac, and respiratory involvement; (2) developmental abnormalities with facial dysmorphism and cognitive impairment inferred from low educational levels and work in specialized environments; and (3) lonely life. Alternatively, women more frequently had cataracts, dysphagia, digestive tract dysfunction, incontinence, thyroid disorder and obesity. Most differences were out of proportion to those observed in the general population. Compared to women, males were more affected in their social and economic life. In addition, they were more frequently hospitalized for cardiac problems, and had a higher mortality rate.

**Conclusion:**

Gender is a previously unrecognized factor influencing DM1 clinical profile and severity of the disease, with worse socio-economic consequences of the disease and higher morbidity and mortality in males. Gender should be considered in the design of both stratified medical management and clinical trials.

## Introduction

Myotonic dystrophy type 1 (DM1 or Steinert´s disease; OMIM 160900) is the most frequent adult muscular dystrophy with a prevalence of about 5/100000 [1, 2]. DM1 clinical manifestations include prominent distal muscle weakness, myotonia and multiorgan involvement mainly affecting the heart, eye, brain, and endocrine system [3]. This autosomal dominant genetic disease is caused by an unstable trinucleotide CTG repeat expansion in the 3’ UTR of the DM protein kinase (*DMPK*) gene [4–7]. Unaffected individuals have 5–37 CTG repeats in this gene which remains stable over generations. In DM1, unstable >40 CTG repeats expansion tends to increase among the different tissues and in successive generations accounting for the anticipation phenomenon [8–10]. The CTG expansion length which correlates inversely with the disease age of onset is currently the only known predictor of some phenotypic manifestations. However, modifier gene or epigenetic factors causing conspicuous variability of DM1 multisystemic involvement in a given individual remain poorly understood.

Of note, large collections of DM1 patients are scant in the literature and usually focus on given manifestations, such as cardiac or endocrine symptoms [11–34]. The gender of the transmitting parent is known to affect CTG size in the offspring, causing preferential transmission of congenital forms by the mother [35–36]. However, gender has not been specifically studied and disease manifestations are considered similar in male and female patients. Here, by a cross-sectional analysis of a large cohort of DM1 patients enrolled in the DM-scope registry and two additional independent nationwide databases we identified the impact of gender on DM1 phenotype’s severity, socio-economic conditions and mortality.

## Materials and Methods

### Study design

#### Cross-sectional analysis of the DM-scope registry

**Fig 1:**
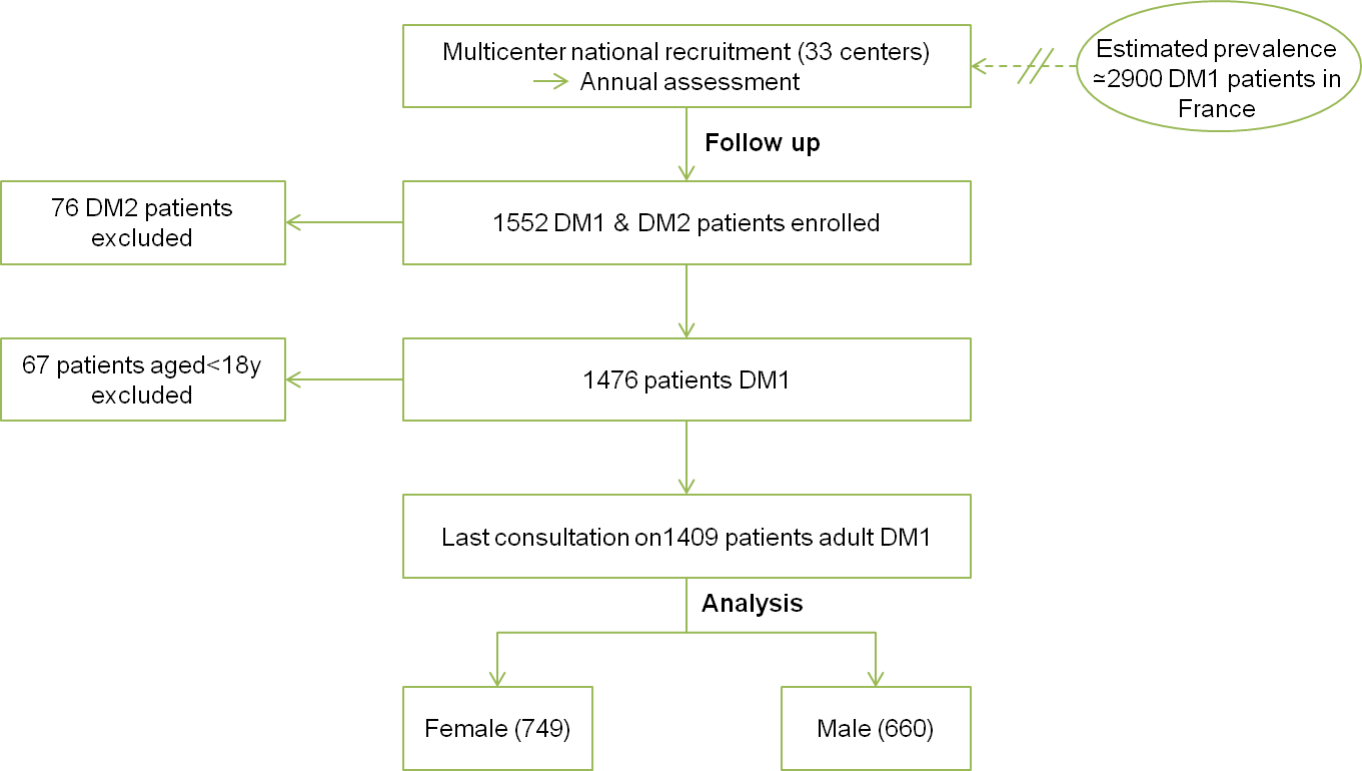
Patient selection of DM-Scope registry.

We first analysed 1409 French adult DM1 patients (>18yrs) of the DM-scope registry (Fig 1), which collects in a standardized form relevant clinical and epidemiological data during routine medical evaluation performed in French neuromuscular reference centers. Analyses focused on two themes: patients’ genotype/phenotype and socio-economic conditions.

#### Complementary study in two nationwide systems

**Fig 2:**
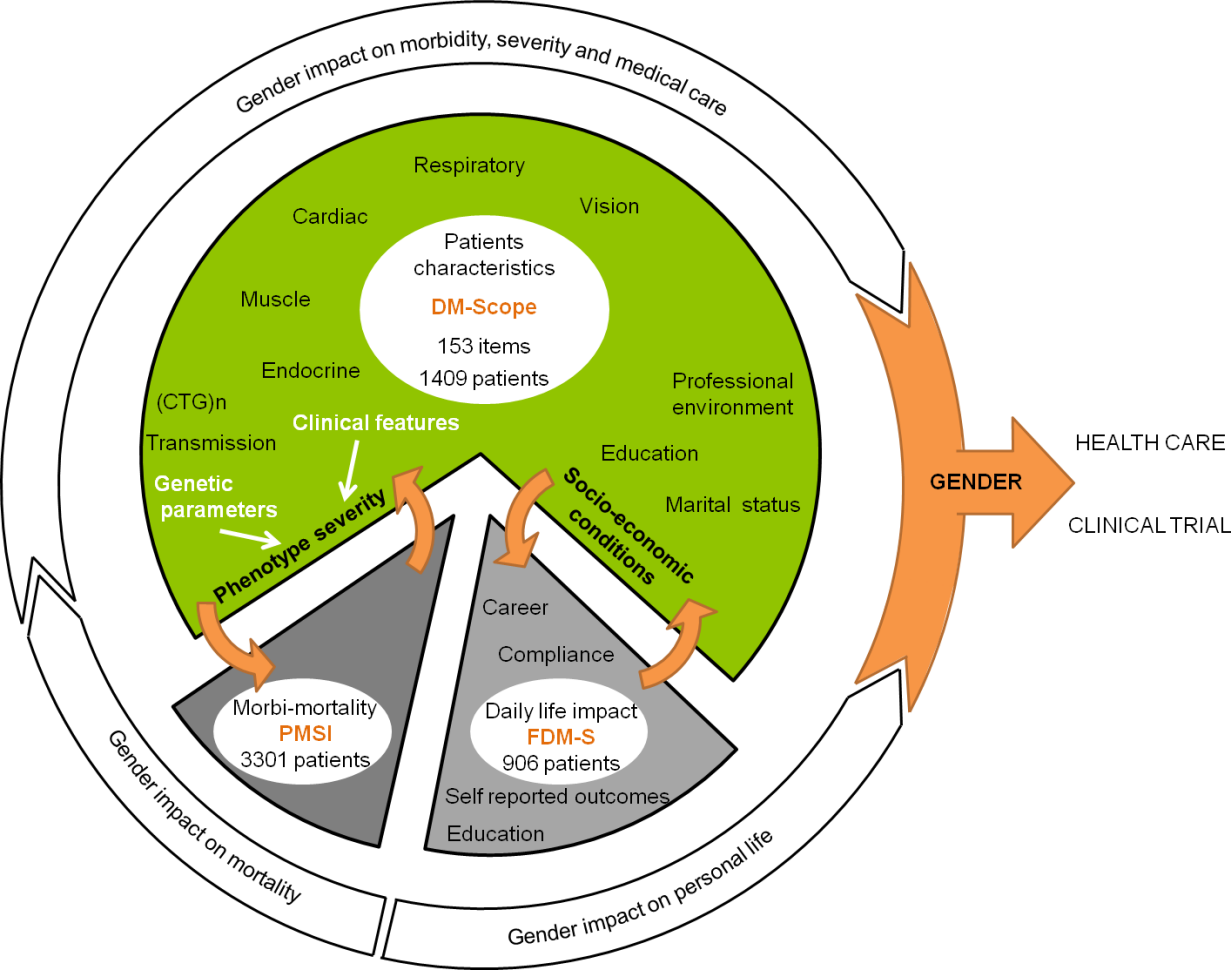
Design of cross-sectional observational study and respective contribution of three national databases: DM-Scope, FDM-S, PMSI Relevant clinical and epidemiological data from French DM1 adult patients (n = 1409), enrolled in DM-Scope registry, were compared to two complementary independent databases, AFM French DM1 survey of patients health and medical care (FDM-S, n = 970 patients) and the National Health Service Database (PMSI, n = 3301), according to similar criteria (age>18y, standardized nationwide collection). Analyses focused on gender effect as a modifying factor of DM1 clinical phenotype, socio-economic status, morbidity and mortality.

We used two other independent national databases (Fig 2) for initial analysis validation and for complementary results (i) the French DM1 patients survey of patients’ health and medical care (FDM-S, n = 970) and (ii) the French National Health Service Database (PMSI—Programme de Médicalisation des Systèmes d'Information, n = 3301) to assess morbidity and mortality. In FDM-S survey, the AFM-Telethon foundation asked DM1 patients (>18yrs) to fulfill questionnaires about their socio-economic status, the main symptoms with their specific impact on the everyday life, medical care and genetic counselling. The PMSI database monitors and harmonizes medical care in hospitals in France. We analysed data collected on French adult DM1 patients (>18yrs) in 2010–2011, with emphasis on history of hospitalizations and death notification. The DM-scope, FDM-S and PMSI databases were approved by the data protection national authority; the DM-scope study was approved by ethics committee and participating patients granted their informed consent. The DM-scope study was approved in 2008 by the CNIL (National Commission on Informatics and Liberty, #1282122). As an information system restricted to standard medical use, no written consent is required by the CNIL and all participating patients received information letter and granted their verbal informed consent. Patient consent is documented in the database and in all centers. This consent procedure has been approved by the national ethic committee CCTIRS (Advisory Committee on Information Processing in Material Research in the Field of Health)

### Definitions and classification

#### Clinical manifestations

Muscle strength was measured using the modified-Medical Research Council (MRC) scale [37] evaluating neck flexors, shoulder abductors, elbow flexors and extensors, wrist extensors, finger flexors, hip flexors, knee flexors and extensors, posterior foot flexors, and plantar flexors muscles. Muscle strength of each muscle group was graded utilizing a modified MRC score that was converted to a 11-point scale (from 0 to 10), instead of 6 point scale. We used the validated muscular impairment rating scale (MIRS) to quantify DM1-related motor impairment [38].

We used the educational level based on International Standard Classification of Education (ISCED) for indirect assessment of cognitive performance.

The following definition criteria were used: “severe myotonia”: time to open hands after contraction ≥3sec; “respiratory insufficiency”: forced ventilation capacity (FVC) <70%; “hypoxemia”: PaO_2_<70mmHg; “hypercapnia”: PaCO_2_>45mmHg “cardiac conduction defects”: ECG showing left hemiblock or bundle branch block; “abnormal ECG”: PR >200ms or QRS >120ms; “somnolence”: Epworth sleepiness scale score >10; “dysphagia”: coughing during eating or drinking more than twice a month and 80 mL-swallowing time test >6 sec [3]; “digestive disorders”: diarrhoea or constipation; “muscle impairment”: muscular impairment rating scale (MIRS) score >3; “couple’s hypofertility”: delay to get the first child >18 months; “overweight/obesity”: body mass index (BMI) >25.

#### Clinical classification

There is no consensus clinical classification of DM1. Besides the widely accepted *congenital myotonic dystrophy*, there are various definitions of childhood-onset DM1. Herein, we classified DM1 into five clinical forms according to the age at onset of first clinical manifestation (adapted from [4]): (1) congenital form: neonatal (mild to severe) hypotonia, respiratory distress, sucking or swallowing difficulties, or skeletal deformities detected at birth or during the first month of life; (2) infantile form: clinical onset from 1 month to 10 years; (3) juvenile form: onset at 11–20 years; (4) adult form: onset at 21–40 years; (5) late onset form: onset after 40 years.

### Statistical analyses

Cross-sectional analysis of the whole DM-scope registry was carried out using data collected in the last medical appointment, prior to July 2013. Categorical variables were compared using the chi-square or Fisher test. Quantitative variables showing non-Gaussian distribution were compared using the non-parametric Mann-Whitney U test. The means of the CTG repeat expansions were compared by the non-parametric Kruskal Wallis test, with the Dunn post-hoc test. Gender differences for several disease-related features were expressed as the risk ratio (RR) of men/women with 95% confidence interval (CI). RR was statistically significant at alpha = 0.05 when 95% CI boundary values did not overlap value 1. P-values <0.05 were considered significant. Statistical analyses were performed using the using R 3.0.2 software (the R Foundation for Statistical Computing, Vienna, Austria).

## Results

### Population characteristics

**Fig 3:**
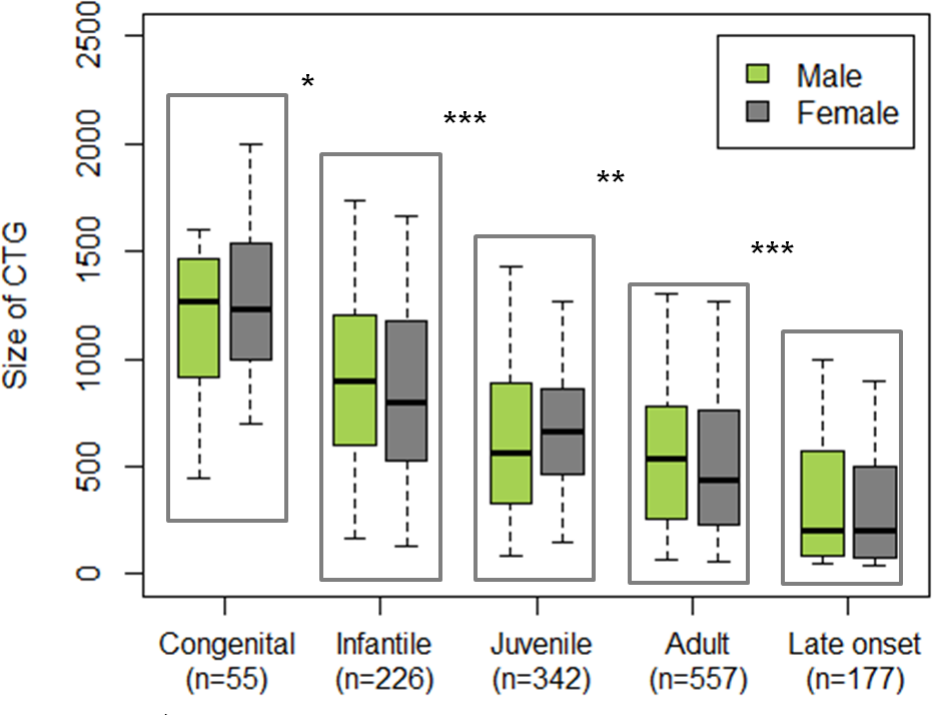
CTG repeat expansion size in male and female individuals according to DM1 clinical forms. Analyses assessed robustness of the conventional age of onset-based classification of DM1 with regard to the triplet expansion size. Inside each clinical form, no difference in CTG expansion size was observed between male and female groups. *Performed tests*: multiple comparison of mean (non parametric Kruskal Wallis test and post hoc test; * p<0.*05*, ** p<0.*001*, ***p<0.*0001)*.

Among the 1409 adult DM1 patients enrolled since 2009 in the DM-Scope registry, a small majority (53.15%) were women, but age, CTG repeats expansion size, age-of-onset, and distribution into the 5 clinical forms were similar between men and women, allowing comparisons of genders in further analyses (Table 1). A strong correlation was found in both men and women between CTG repeat expansion size and the different clinical forms (Fig 3), supporting the robustness of the classification based on the age-of-onset.

**Table 1:** Characteristics of patients in the DM-scope registry.

	Mean age of patients in yrs (SD)	Mean age of onset (SD)	Mean CTG expansion (SD)	Congenital form in %	Infantile form in %	Juvenile form in %	Adult form in %	Late onset form in %
**Female (749)**	42.65 (12,8)	24.53 (14)	647.72 (452)	3.59	15.72	25.66	42.21	12.83
**Male (660)**	42.38 (13.4)	23.72 (15)	628.9 (433)	4.59	17.72	24.68	39.72	13.29
**Overall (1409)**	42.52 (13)	24.15 (14)	639.1 (443)	4.05	16.65	25.2	41.05	13.04
	*NS*	*NS*	*NS*	*NS*	*NS*	*NS*	*NS*	*NS*

### Gender of transmitters and severity

As a general rule, repeat expansions were longer in case of maternal inheritance (mean 804 ±507) *vs*. (600 ±393) in case of paternal transmissions, p<0.0001). Surprisingly, congenital DM1 onset was not exclusively associated with maternal inheritance (91% maternal inheritance *vs* 9% paternal inheritance). Infantile forms were equally transmitted by mothers (50%) and fathers (50%) (Table 2). Maternally inherited infantile forms showed longer repeat expansions (1051 (±401) *vs* 760 (±376), p<0.0001). more severe cognitive impairment (ISCED≤3, p = 0.01; specialized educational environment, p = 0.005) and developmental abnormalities (facial dysmorphism, p<0.05) than paternally inherited ones. Unexpectedly for an autosomal dominant disorder, juvenile, adult and late onset forms of DM1 were preferentially transmitted by fathers (67, 68 and 63% of paternal transmission, respectively) (Table 2).

**Table 2:** Gender effect on disease transmission and CTG expansion.

Parental transmission	Congenital form	Infantile form	Juvenile form	Adult form	Late onset form	Overall
**Maternal transmission %**	91	50	28	33	32	37
**Maternal transmission: mean CTG expansions (SD)**	1337 (684)	1051 (401)	784 (369)	617 (393)	294 (310)	804 (507)
**Paternal transmission: mean CTG expansions (SD)**	1190 (711)	760 (376)	668 (399)	538 (359)	346 (340)	600 (393)
	*NS*	*p<0*.*0001*	*p<0*.*05*	*NS*	*NS*	*p<0*.*0001*

### Gender of patients and clinical symptoms

**Fig 4:**
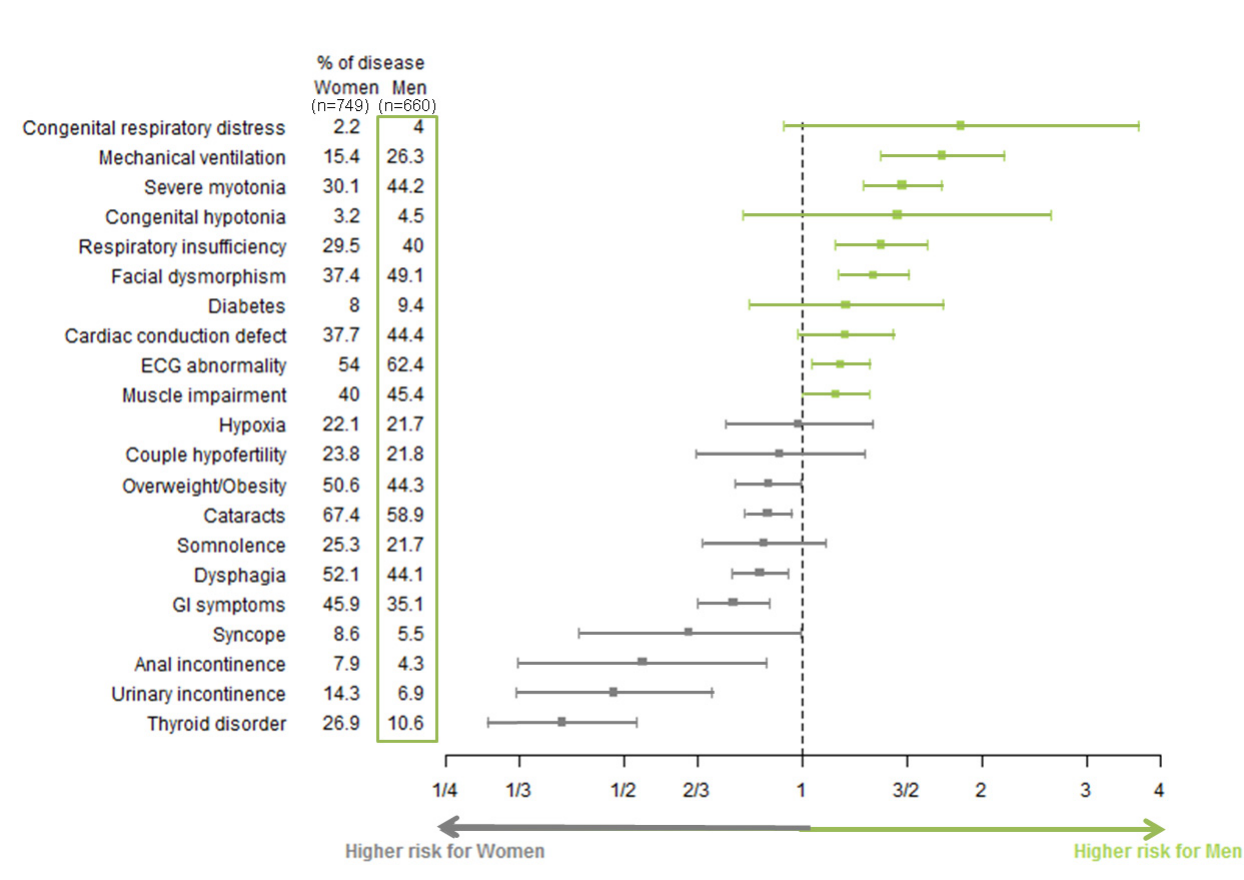
Gender impact on severity of symptoms expressed as risk ratio on 95% confidence interval. This diagram represents the gender relative risk ratio value for each symptom with its 95% confidence interval (segment). A risk ratio is significant if the confidence interval does not cross the vertical line at value 1. The width of confidence interval depends on estimate standard deviation and consequently on observations number.

The frequency of some symptoms differed, to various extents, between men and women. To illustrate the influence of gender on each symptom, relative risks for men and women were calculated and expressed in form of a forest plot (Fig 4). Men more frequently had developmental abnormalities, severe myotonia, severe cardiac and respiratory involvement and muscle weakness, as assessed by MRC testing (p = 0.001). Women had more frequently cataracts, dysphagia, digestive tract dysfunction, incontinence, thyroid disorder and obesity.

By reference to the general French population, DM1 clinical manifestations were ascribed to 3 subgroups: (1) manifestations virtually not encountered in the general population but more frequent in a given gender in DM1: facial dysmorphism, myotonia, and delayed conduction at ECG in males; and dysphagia in females; (2) manifestations more frequent in a given gender in the French population but not in the DM1 population (similar frequency in DM1 males and females): higher risk ratio of diabetes in French males [39] was reduced and not significant in both genders in DM1 patients (RR of diabetes in males of the DM1 cohort 1.18 *vs*. 1.42 in the general population); (3) manifestations more prevalent in a given gender in both the general and DM1 population, with an accentuated gender effect in DM1 for thyroid disorders and obesity (RR of thyroid disorders in females of the DM1 cohort 2.15 vs. 1.12 in the general population; RR of obesity in females of the DM1 cohort 1.58 *vs*. 1.10 in the general population [40,41]).

### Gender of patients and socio-economic conditions

Comparative analyses between men and women revealed also a different impact of DM1 on social and economic conditions. Men more often had lonely life styles (single: 34% in women *vs*. 60% in men, p<0.0001), low education level (ISCED≤3: 62 in women *vs*. 68% in men p<0.05) and work in protected environments (30 *vs*. 49%, p<0.05). Regarding the marital status, males living alone were found markedly enhanced in DM1 (RR of single status in males of the DM1 cohort is 1.74 *vs* 1.05 in the general population). Lower educational levels also more prevalent in males was proportionate in the DM1 and general population (RR of ISCED≤3 in males of the DM1 cohort is 1.10 *vs* 1.16 in the general population [42])

### Substantiation of gender impact on DM1 using FDM-S and PMSI databases

The FDM-S survey and the PMSI hospitalization database included 56.4% and 49.8% female DM1 patients, respectively, and this was considered in the analytical methods to limit bias. In the three cohorts, DM1 patients were comparable for mean age (42.5 yrs (±13.1) in DM-Scope *vs*. 45.5 (±12.1) in FDM-S *vs*. 47.9 (±14.3) in PMSI). Comparison for CTG repeat expansion was not performed (data not available in FDM-S and PMSI).

**In FDM-S (n = 970),** men and women showed similar medical compliance, consulting as frequently the main specialists (cardiologist, pneumonologist, neurologist, and psychologist), at the exception of ophthalmologists more often met by women (p<0.03). Analysis of the more disabling symptoms reported in FDM-S confirmed gender-dependent differences detected in the DM-scope registry (Table 3). Men presented more frequently cardiac arrhythmia and respiratory insufficiency. Half of them complained of elocution difficulties, facial weakness (p<0.0001), and writing difficulties (p<0.001) related to either severe myotonia (p<0.0001). Women more frequently reported cataracts. According to the DM-scope registry, gender does not affect somnolence or diabetes. However, in FDM-S, women did not complain of dysphagia more often than men.

**Table 3:** Comparison of most disabling symptoms in FDM-S to DM-Scope registry related findings.

**FDM-S**		**Cardiac conduction defects**	**Respiratory insufficiency**	**Cataracts**	**Elocution difficulties**	**Writting difficulties**	**Somnolence**	**Diabetes**	**Dysphagia**
	**Male**	45%	34%	47%	51%	50%	40%	9%	29%
	**Female**	37%	23%	59%	32%	34%	44%	7.2%	25%
		*p<0*.*001*	*p<0*.*001*	*p<0*.*0001*	*p<0*.*0001*	*p<0*.*001*	*NS*	*NS*	*NS*
**DM-Scope**		**ECG abnomalities**	**Respiratory insufficiency**	**Cataracts**	**Facial dysmorphism**	**Severe myotonia**	**Somnolence**	**Diabetes**	**Dysphagia**
	**Male**	44.4%	40%	58.9%	49%	44%	21.7%	9.4%	44.1%
	**Female**	37.7%	29.5%	67.4%	37%	30%	25.3%	8%	52.1%
		*p<0*.*01*	*P<0*.*001*	*p<0*.*01*	*p<0*.*0001*	*p<0*.*0001*	*NS*	*NS*	*p<0*.*01*

FDM-S survey also confirmed the impact of gender on socio-economic parameters. It included nearly half of single men (60% in the DM-scope cohort), and bachelorhood was considered as a serious life-impacting problem by 30% of DM1 males. Educational difficulties were attributed to slowness (p<0.05) and were more often responsible for educational switch in men (p<0.05). Men also experienced career changes more often (p<0.05).

**PMSI database (n = 3301) analysis** revealed gender impact on DM1 morbi-mortality. Hospitalizations of DM1 patients where compared to 11304 hospitalizations of gender groups of similar age during the same 2010–2011 period. DM1 males patients had higher hospitalization rate for cardiac conduction defect (p = 0.003) or respiratory insufficiency (p = 0.03), whereas women were preferentially admitted for visual difficulties (p = 0.001) and endocrine or metabolic dysfunction (p<0.0001). Analysis of death declarations indicated a higher mortality rate in men (p = 0.009).

## Discussion

Our study revealed the multidimensional influence of gender in DM1. First, maternal inheritance was associated with longer repeat expansions and more severe phenotype, as previously reported [5, 6]. This has been attributed to marked DNA instability in the female germ cell lineage allowing additional triplets insertion during oogenesis [43]. Such instability also results to an anticipation in case of maternal inheritance, a phenomenon corresponding to earlier onset and more severe symptoms observed in successive generations [10]. Surprisingly, and in contrast to the general assumptions, we observed that fathers transmitted up to 9% of neonatal onset (mild or severe) forms and 50% of infantile forms, especially those with lower cognitive impairment. Another unexpected observation was that only a minority of overall DM1 patients (37%) had maternal inheritance, which is most unusual for an autosomal dominant inherited disease. It probably results from increased miscarriage and perinatal lethality observed in female DM1 transmitters.

The second gender difference implied an unequal prevalence of several DM1 signs and symptoms in men and women. These differences could not be accounted for overall quantitative male-to-female disproportion in our study population (considered in all statistical analysis), or for the age and genotype differences between the two groups. Men tended to have more obvious classical DM1 symptoms, combining cognitive impairment, marked myotonia, cardiac and respiratory involvement whereas women had more extra-muscular and late onset manifestations, less suggestive of DM1, such as cataracts, obesity, dysthyroidism, GI symptoms and sphincter dysfunction. The most poorly symptomatic patients were women, implicating occasional hidden DM1 transmissions by undiagnosed female mutation carriers. In practice, the sex-related differential risks of developing specific manifestations may require sex-orientated care management, which should be specifically adapted for men (at higher risk of mechanical ventilation, respiratory failure or cardiac conduction defects, which could provide more frequent hospitalization and increased mortality according PMSI database) as well as for women (at higher risk of dysthyroidism, obesity, sphincter dysfunction, and cataracts). This gender disproportion suggests that women could be more careful with their own health. This is underlined by FDM-S survey showing a similar number of annual routine visits to the cardiologist and pneumonologist for both genders, despite male have more cardiac and respiratory involvement, which should prompt more regular medical care. Altogether, the results highlight the importance of a greater awareness about preventive medical care in DM1 male individuals.

The major strengths of this study include (i) the unprecedented number of patients with genetically confirmed DM1 included in the DM-scope cohort, (ii) the prospective and standardized collection of data in the registry performed by expert physicians, (iii) the single country-based enrolment of patients allowing comparisons with the general population, and (iv) the validation of important results by analysis of two other national DM1 sources (FDM-S, PMSI). Similarly described in the litterature, cross sectional studies discussed in our work are inappropriate for the analysis of age-dependant symptoms in a given clinical form. This point deserves a follow-up study using complex *ad hoc* statistical approaches.

The exact mechanisms underlying gender-dependent differences remain unknown. More diffusely severe myopathy in male patients assessed by MRC testing was reminiscent of some other muscular diseases of autosomal inheritance, such as FSHD, hypokalemic periodic paralysis (HypoPP) or *ANO5* mutation-related myopathy [44]. It has been experimentally established that both genetic background [45] and male gender may influence the severity of weakness in mouse autosomal muscular dystrophy. Male and female skeletal muscles display considerable differences in their metabolic properties and gene expression patterns [46,47]. In congenital myotonia, sexual steroid hormones influence severity of myotonia, reflecting testosterone modulation of CLCN1 chloride channel activity [48]. In cardiovascular diseases, it has been described a possible early-life sex-dependent vulnerability to oxidative stress [49]. Other gender-related cellular differences can be considered, as recently proposed [50].

## Conclusion

In conclusion, the present study identified gender as a previously unrecognized factor influencing DM1 manifestations with sex-related distinctive clinical profiles and severity. Worse socio-economic consequences of the disease and higher morbidity and mortality were observed in DM1 males. Gender of DM1 patients should be considered in the design of both stratified medical management and clinical trials.

## Abbreviations

AFM: “Association Française contre les myopathies”
BMI: Body mass Index
CI: Confidence Interval
DM1: Myotonic Dystrophy type I
FDM-S: French DM1 patients’ survey
FSHD: Facioscapulohumeral Muscular Dystrophy
FVC: Forced Ventilated Capacity
HypoPP: Hypokalemic Periodic Paralysis
ISCED: International Standard Classification of Education
MIRS: Muscular Impairment Rating Scale
MRC: Medical Research Council Scale
PMSI: French National Health Service Database
RR: Risk Ratio
UTR: Untranslated region
